# Viewing entrepreneurship through a goal congruity lens: The roles of dominance and communal goal orientations in women’s and men’s venture interests

**DOI:** 10.3389/fpsyg.2023.1105550

**Published:** 2023-03-22

**Authors:** Abigail Folberg, Tara Goering, Lindsey Wetzel, Xiaoming Yang, Carey Ryan

**Affiliations:** ^1^Department of Psychology, University of Nebraska Omaha, Omaha, NE, United States; ^2^Department of Psychological Sciences, Ball State University, Muncie, IN, United States; ^3^Department of Marketing and Entrepreneurship, University of Nebraska Omaha, Omaha, NE, United States

**Keywords:** gender, entrepreneurship, gender stereotypes, agentic and communal goal orientations, entrepreneurship education

## Abstract

The objective of this research was to examine gender differences in entrepreneurial venture interests drawing on goal congruity theory, which posits that people adopt gender-stereotypic goal orientations in response to social pressures to conform to traditional gender roles. Aspiring entrepreneurs (*N* = 351) first wrote about what they believed made an entrepreneur successful. They then completed measures of agentic and communal goal orientations (i.e., male and female stereotypic orientations, respectively) and indicated their interests in starting ventures in stereotypically feminine (e.g., salon), masculine (e.g., auto-repair) and science, technology, engineering, and mathematics (STEM; e.g., software developer) ventures. Analysis of open-ended responses demonstrated that participants ascribed more agentic and, specifically, more dominance attributes to entrepreneurs than communal attributes (e.g., warmth). Bifactor structural equation modeling indicated that, as expected, agentic goal orientations included dimensions of competence, self-direction, and dominance orientations; communal goal orientations were unidimensional. Further, as expected, dominance and communal orientations partially accounted for gender differences in all three career types. We discuss implications for entrepreneurial education and practice from a goal congruity perspective and the use of bifactor modeling to improve the measurement of goal orientations.

## 1. Introduction

Women entrepreneurs have been regarded as “blemished men” (c.f., Marlow, 2002; Ahl, 2006), who lack qualities, such as risk-taking, competitiveness, and ambition (Laguía et al., 2019; Wilhau and Karau, 2021), that are consistent with stereotypes of men (vs. women; Eagly et al., 2000). These stereotypes are associated with decreases in women’s entrepreneurial interest, self-efficacy (BarNir, 2021), and growth expectations (Martiarena, 2020) and contribute to gender disparities in funding (Brush et al., 2019; Laguía et al., 2019). Research is, thus, needed to identify factors that may decrease gender disparities in entrepreneurial interests and persistence.

In the present research, we draw on goal congruity theory (Diekman et al., 2010), which has been instrumental in understanding gender differences in science, technology, engineering, and mathematics (STEM) career interests and persistence (see Diekman et al., 2017, for a review). We examine among a crowd-sourced sample of aspiring entrepreneurs, whether successful entrepreneurship is perceived as requiring dominance, but not qualities typically ascribed to women. We also examine the role of agentic and communal goal orientations (i.e., male-stereotypic and female-stereotypic goals, respectively) in participants’ entrepreneurial interests, answering calls to apply goal congruity theory more to non-STEM fields (Diekman et al., 2020). Finally, we examine subdimensions of agentic and communal goal orientations, extending research examining subdimensions of gender stereotypes (Hentschel et al., 2019; Folberg et al., 2022) and stereotype-related constructs (Folberg et al., 2020).

### 1.1. Gender and entrepreneurship

Stereotypes of entrepreneurs generally reflect stereotypes of men (Gupta et al., 2005; Laguía et al., 2019; Wilhau and Karau, 2021)—societal assumptions that are also present in entrepreneurial research (Marlow, 2002; Ahl, 2006) and education (Gupta et al., 2019). One high school entrepreneurship curriculum (Wagner et al., 2021), for example, suggested that good entrepreneurs, “want financial success,” “take risks,” “are independent,” and “have a need to achieve”—qualities associated with men more than women (Eagly et al., 2000). The training failed to identify characteristics stereotypic of women (e.g., likes working with others) as leading to entrepreneurial success, consistent with other work suggesting stereotypically feminine traits are viewed as incompatible with entrepreneurship (e.g., Ahl, 2006).

Yet, business owners’ descriptions of their work suggests that stereotypically feminine qualities, such as being highly relationship-oriented, working with others (Malach-Pines and Schwartz, 2008; Laguía et al., 2019), and being “sympathetic,” and “aware of the feelings of others” (Gupta and Fernandez, 2009) are necessary for success. Entrepreneurship also provides people with opportunities to enrich their communities (Wilhau and Karau, 2021) and flexible work-life balance (Walker et al., 2008)—qualities women often value more than do men (e.g., Diekman et al., 2017). Thus, understanding—and potentially changing—the perceived incongruity between stereotypes of women and qualities of successful entrepreneurs may increase women’s entrepreneurial interest. Goal congruity theory provides a useful framework for guiding these efforts.

### 1.2. Goal congruity theory and entrepreneurship

According to goal congruity theory (Diekman et al., 2010), gender stereotypes stem from women and men’s distribution into social roles (Eagly et al., 2000). Women are perceived as more communal because they occupy roles (e.g., childcare provider) that require them to exhibit communal behaviors, such as warmth, whereas men are perceived as more agentic because they occupy roles (e.g., business leader) that require them to exhibit agentic behaviors, such as dominance. Women and men seek to align themselves with socially prescribed gender roles (Prentice and Carranza, 2002) and, thus, develop gender-role congruent goals, which facilitate gender differences in career interests (Diekman et al., 2010, 2017, 2020; Folberg et al., 2020). Women more strongly endorse communal goals, which predict greater interest in female-stereotypic (FST; e.g., nurse) careers and less interest in STEM careers. In contrast, men more strongly endorse agentic goals, which predict greater interest in male-stereotypic (MST; e.g., doctor) careers and potentially STEM careers (Diekman et al., 2010, 2017). Thus, women and men entrepreneurs might similarly exhibit gender differences in goal orientations, which may facilitate their interest in stereotype-consistent ventures (Wilhau and Karau, 2021).

Research on stereotypes and goal orientations has traditionally treated agency and communion as two unidimensional constructs (Diekman et al., 2010; Eagly et al., 2020), although both may comprise distinct subdimensions (Hentschel et al., 2019; Folberg et al., 2020, 2022). Further, not all dimensions of gender stereotypes and goal orientations are useful for understanding gender differences, likely because aspects of agency and communion are judged differently. For example, perceived dominance drives perceptions of gender differences in agentic traits (Hentschel et al., 2019; Folberg et al., 2022), and women are penalized for displaying dominance, whereas men benefit from displaying dominance (Rudman and Glick, 2001; Okimoto and Brescoll, 2010). Further, women are encouraged to be communal, warm, and nurturing (Prentice and Carranza, 2002), men are often encouraged to eschew communal qualities (Vandello et al., 2013), and men perceived as communal may lose status (Rudman and Glick, 2001).

Women and men might therefore be perceived as most distinct in communion and dominance. Indeed, Folberg et al. (2022), using bifactor structural equation modeling, found that gender differences in self- and group-stereotypes most consistently emerged in communion and dominance. Further, gender differences in communion and dominance self-stereotypes were stronger among individuals who viewed their gender identity as more salient. Other dimensions of agency, including competence, self-direction, and global measures of agency, did not reliably yield corresponding gender differences. Thus, perceptions that entrepreneurship is inherently masculine (Laguía et al., 2019; Wilhau and Karau, 2021) seem likely to be driven by dominance more than other types of agentic traits. Indeed, attributes ascribed to entrepreneurs typically reflect dominance, for example, liking power (Laguía et al., 2019), self-promotion (Gupta et al., 2019), and being competitive (Díaz-García and Jiménez-Moreno, 2010).

Folberg et al. (2020) also used bifactor modeling to show that communal goal orientations were unidimensional, but agentic goal orientations comprised a global competence dimension, and domain-specific dominance (i.e., a desire to have status or power over others), and self-direction goal orientations (i.e., a desire to pursue empowerment and independence) (See Table 1). Gender differences emerged only for communal and dominance goal orientations. Further, as expected, communal goal orientations facilitated women’s greater interest in FST careers, whereas dominance goal orientations facilitated men’s greater interest in MST and STEM careers. Neither self-direction nor global competence or self-direction goals explained gender differences in career interest.

**Table 1:** Dimensions of goal orientations.

Goal orientation	Definition	Items
Communal	Goals relating to being prosocial and emotional intimacy	Serving humanity, Working with people, Attending to others, Intimacy, Helping others, Serving community, Caring for others, Connection with others, Spiritual rewards
Agentic	Goals relating to showing assertiveness, competence, and self-direction	Power, Achievement, Self-promotion, Individualism, Success, Self-direction, Demonstrating competence, Competition, Recognition, Financial rewards, Mastery, Independence, Focus on the self, Status
Dominance	Goals relating to having power over others	Power, Self-promotion, Competition, Recognition, Financial rewards, Status
Self-direction	Goals relating to empowerment and independence	Individualism, Self-direction, Independence, Focus on the self

Communal and dominance goals may, therefore, similarly help explain gender differences in venture interests. We view the focus on dominance goals as particularly important because goal congruity theory has traditionally focused almost exclusively on the role of communal goals in STEM interest and persistence (Diekman et al., 2017, 2020). The role of agentic goals in facilitating gender differences in career interests is less well-examined (Diekman et al., 2017), likely because misspecifications of agency and agentic goals as unidimensional obscured the substantial effects of dominance goals and stereotypes (Folberg et al., 2020, 2022).

### 1.3. The present study

We assessed perceptions of entrepreneurs, goal orientations, and venture interests among self-identified aspiring entrepreneurs, expecting that people would ascribe to successful entrepreneurs more traits related to agency (vs. communion). We further expected that among agentic traits, participants would ascribe more traits relating to dominance (vs. competence and independence), consistent with Folberg et al. (2020, 2022).

We used bifactor modeling to assess whether communal and dominance goals (vs. other agentic goals) partially account for gender differences in venture interests. We expected that women would have stronger communal goal orientations and would thus express more interest in starting ventures in female-stereotypic domains (e.g., salon/spa owner) and less interest in starting ventures in STEM (Diekman et al., 2010, 2011, 2017; Folberg et al., 2020) and other male-stereotypic domains (e.g., auto-repair; Diekman et al., 2010, Folberg et al., 2020). In contrast, we expected that men would have stronger dominance goal orientations and thus exhibit greater interest in male-stereotypic and STEM ventures and less interest in female-stereotypic ventures. Finally, consistent with previous work (Folberg et al., 2020), we expected communal and dominance goal orientations to account for gender differences in entrepreneurial venture interests.

## 2. Method

### 2.1. Participants

This research was approved by the IRB at the University of Nebraska Medical Center and conforms to the ethical standards for research involving human participants. Data and stimulus materials are available at https://osf.io/dazy9/?view_only=229a10866c7b4afaace38dce69b3dfd8.

Participants who had expressed interest in starting an entrepreneurial venture on a screening survey (*N* = 351) were recruited from Prolific for a study examining entrepreneurial intentions. Participants were paid $1.75USD each. The sample size is consistent with guidelines for structural equation modeling (Kline, 2016).

Approximately half (49.9%) of participants identified as women and half (50.1%) as men. Participants identified as White (59.0%), Middle Eastern/Arab (14.2%), Asian/Pacific Islander (12.5%), Black (1.3%), Latinx (8.5%), multi-racial/ethnic (6.6%), and other (1.4%). Fewer than 1% of participants identified as Native American or East Indian. (A question assessing participant age was inadvertently omitted.) Participants’ highest level of completed education was a high school degree or its equivalent (e.g., GED; 8.6%), some undergraduate education (i.e., college; 8.6%), an associate’s degree (7.7%), college or post-graduate education (e.g., master’s degree, professional degree or Ph.D.; 57.3%), or did not complete high school (<1%).

### 2.2. Procedure

Participants were first asked to describe a successful entrepreneur (open-ended). They were then asked to describe the venture they wanted to start. Participants intended to start a variety of ventures, including tech, restaurants/food industry, and retail (see Supplementary materials).

Next, participants indicated how likely they would be to pursue a venture in each of 18 domains (i.e., auto repair, lawn care/landscaping, financial advising, environmental engineering, florist, childcare, salon/spa, cleaning services, fashion boutique, interior design, event planning, restaurant, bar/pub, public relations, computer/cell phone repair, IT consulting, forensic science, and software development) on a 1 (*Very Unlikely*) to 7 (*Very Likely*) scale.

Embedded within these domains were 13 target domains. Six were stereotypic of women (FST; florist, childcare, salon/spa owner, cleaning services, event planning, interior design), four were stereotypic of men (MST; construction, auto repair, lawncare/landscaping, financial advising), and three were STEM careers (computer/cell phone repair, IT consulting, software development). In a separate sample of 25 women and 25 men, we confirmed that FST (vs. MST and STEM) careers were perceived as more commonly performed by women than men; estimated percentages of women and men in each field were also strongly correlated with actual percentages of women and men in each field provided by the U.S. Bureau of Labor Statistics (see Supplementary materials) Thus, participants’ perceptions of the distribution of women and men in FST, MST, and STEM fields accurately reflected the actual distribution of women and men in each career domain (Ryan, 2002).

Next, participants completed Diekman et al.’s (2010) 23-item measure of agentic and communal goal orientations. (See Table 1 for the list of items.) Participants indicated how important each goal was to them on a 1 (*Not at all Important*) to 7 (*Extremely Important*) scale.

#### 2.2.1. Analysis of open-ended responses

We searched for the agentic and communal attributes examined by Folberg et al. (2022), who identified attributes used across 21 person perception studies (Abele et al., 2016) and additional attributes commonly used in gender stereotyping research. Twenty-seven attributes assessed agency, including seven self-direction attributes (i.e., desire responsibility, independent, self-reliant, emotionally stable, self-directed, self-focused, and individualistic), 13 dominance attributes (i.e., ambitious, assertive, can make decisions easily, superior, have leadership abilities, never give up easily, purposeful, self-confident, stand up under pressure, aggressive, competitive, courageous, dominant), and seven competence attributes (i.e., capable, clever, competent, efficient, intelligent, persistent, creative). Nineteen attributes assessed communion, including 11 warmth attributes (i.e., affectionate, caring, empathetic, friendly, helpful, warm, emotional, kind, sensitive, sympathetic, intuitive), and eight morality attributes (i.e., considerate, fair, just, reliable, trustworthy, honest, compassionate, and moral). Morality attributes are more commonly assessed in research on person perception than in gender stereotyping research (e.g., Hentschel et al., 2019; Folberg et al., 2020, 2022); Diekman et al. (2010) measure of goal orientations contains no items assessing morality.

We then examined whether agentic (specifically dominance) versus communal attributes were mentioned more frequently, consistent with expectations and literature associating entrepreneurship with agency (e.g., Laguía et al., 2019). We also explored whether differences depended on participant gender.

#### 2.2.2. Quantitative analyses

We used bifactor modeling (Morin et al., 2016; Rodriguez et al., 2016) to examine the factor structure of goal orientations. In bifactor modeling, global and domain-specific factors are estimated simultaneously, allowing researchers to partition item-level variation into variation accounted for by global factors, domain-specific factors, and error. If items load onto a global factor, but not on domain-specific factors, the measure is assumed to be unidimensional. Bifactor modeling also allows researchers to simultaneously examine whether effects emerge in both global and domain-specific factors, which is not possible using hierarchical factor analysis, first-order factor analysis, or composite measures (Morin et al., 2016).

Models were estimated using full-information maximum likelihood estimation (FIML) with robust standard errors (MLR estimation) and geomin rotation in M*plus* version 8.3 (Muthén and Muthén, 2017). Values of the comparative fit index (CFI) and Tucker-Lewis Index (TLI) exceeding 0.90 and 0.95 indicated adequate and excellent model fit, respectively; values of the root mean-square error of approximation (RMSEA) below 0.08 and 0.06 indicated adequate and excellent model fit, respectively (Hu and Bentler, 1999). Values of the standardized root mean residual (SRMR) below 0.08 also indicated adequate model fit (Asparouhov and Muthén, 2018).

Two indices assessed the fit of the bifactor model (Rodriguez et al., 2016). Omega hierarchical (ωH) is the proportion of item-level variation explained by the global factor across a set of items. Estimates that exceed 0.80 suggest unidimensionality. Omega hierarchical for the subscale (ωHS) indicates the amount of unique item-level variation accounted for by a domain-specific factor across its item indicators over and above the global factor; high ωHS estimates suggest multidimensionality (Rodriguez et al.). Finally, we estimated a structural model that included the direct effects of gender and goal orientations on venture interests and the indirect effects of gender on venture interests *via* goal orientations.

## 3. Results

### 3.1. Analysis of open-ended responses

Table 2 summarizes the frequencies with which dominance, competence, self-direction, warmth, and morality traits were ascribed to successful entrepreneurs. As expected, participants ascribed more agentic than communal traits, *χ*^2^(1) = 98.05, *p* < 0.001. Further, participants were most likely to ascribe dominance traits, followed closely by competence; self-direction was least frequent, *χ*^2^(2) = 67.31, *p* < 0.001. Findings did not depend on participant gender, *p*s > 0.140.

**Table 2:** Number of agentic and communal attributes ascribed to successful entrepreneurs by participant gender.

Attribute type	Women (*n* = 175)	Men (*n =* 176)	Total (*N* = 351)
Agency	70	60	130
Dominance	24	35	59
Competence	32	19	51
Self-Direction	14	6	20
Communion	8	4	12
Warmth	3	2	5
Morality	5	2	7

### 3.2. Quantitative analyses

Estimates of item skew and kurtosis fell within recommended guidelines (Kline, 2016) and fewer than 2% of cases had missing data. The Online Supplement includes item-level descriptive statistics and a description of the measurement models of venture interests and goal orientations.

As expected, our final bifactor CFA measurement model of goal orientations comprised a unidimensional communal goal orientations factor, a global competence orientation factor, and two agentic goal orientation subdimensions: dominance and self-direction. The final measurement model (Table 3) also included FST, MST, and STEM career interests; all factors exhibited good reliability as measured by McDonald’s Omega (McDonald, 1999). Consistent with Folberg et al. (2020), communal goal orientations were associated with stronger global competence goal orientations and weaker dominance goal orientations. Communal goal orientations were also associated with stronger FST venture interests. Global competence goal orientations were weakly associated with greater FST venture interests. Dominance goal orientations were associated with greater interest in all three types of ventures, which were positively interrelated, *χ*^2^(378) = 633.584, CFI = 0.931, TLI = 0.921, RMSEA = 0.044, 90%CI[0.038, 0.050], SRMR = 0.053.

**Table 3:** Standardized factor loadings, reliability estimates, and correlations among latent factors for the final measurement model.

Item	Goal orientations				Career interests		
	Communal ω = 0.92	Global Agentic (Competence) ωH = 0.76	Dominance ωHS = 0.43	Self-direction ωHS = 0.37	STEM ω = 0.86	MST ω = 0.82	FST ω = 0.83
C1. Serving humanity	0.76						
C2. Working with people	0.65						
C3. Attending to others	0.75						
C4. Helping others	0.88						
C5. Serving community	0.83						
C6. Caring for others	0.84						
C7. Connection with others	0.73						
A1. Achievement		0.76					
A2. Success		0.62					
A3. Demonstrating skill/ competence		0.70					
A4. Mastery		0.71					
D1. Power		0.43	0.54				
D2. Self-promotion		0.41	0.40				
D3. Competition		0.44	0.40				
D4. Recognition		0.56	0.49				
D5. Status		0.48	0.65				
S1. Individualism		0.32		0.54			
S2. Self-direction		0.61		0.37			
S3. Independence		0.51		0.55			
STEM1. Computer/Cell Phone Repair					0.75		
STEM2. IT Consulting					0.93		
STEM3. Software Development					0.78		
MST1. Construction						0.77	
MST2. Auto Repair						0.82	
MST3. Lawn Care/Landscaping						0.73	
FST1. Child Care							0.56
FST2. Salon/Spa							0.74
FST3. Interior Design							0.78
FST4. Event Planning							0.71
FST5. Florist							0.70
	1	2	3	4	5	6	7
Goal Orientations							
1. Communal							
2. Global Competence	0.57***						
3. Dominance	−0.17*	0.00					
4. Self-direction	−0.15	0.00	0.00				
Career Interests							
5. STEM	−0.02	0.00	0.27***	0.07			
6. MST	0.05	0.02	0.34***	0.00	0.45***		
7. FST	0.22***	0.15*	0.21***	0.09	0.21***	0.56***	

*N* = 351. ω = McDonald’s (1999) omega, ωH = omega hierarchical for the global factor, and ωHS = omega-hierarchical for the domain-specific factors. All factor loadings are significant, *p* < 0.001.

***p < 0.001; **p < 0.01; *p < 0.05.

Correlations among composite measures, Cronbach’s alphas, and mean differences in composite measures by gender are provided in the Online Supplement. However, as composite measures confound variation due to the global construct, domain-specific constructs, and random error (Rodriguez et al., 2016; Folberg et al., 2020), they are less accurate. We, thus, strongly encourage readers to rely on the statistics provided in Table 3.

### 3.3. Relationships of goal orientations with venture interests

**Figure 1:**
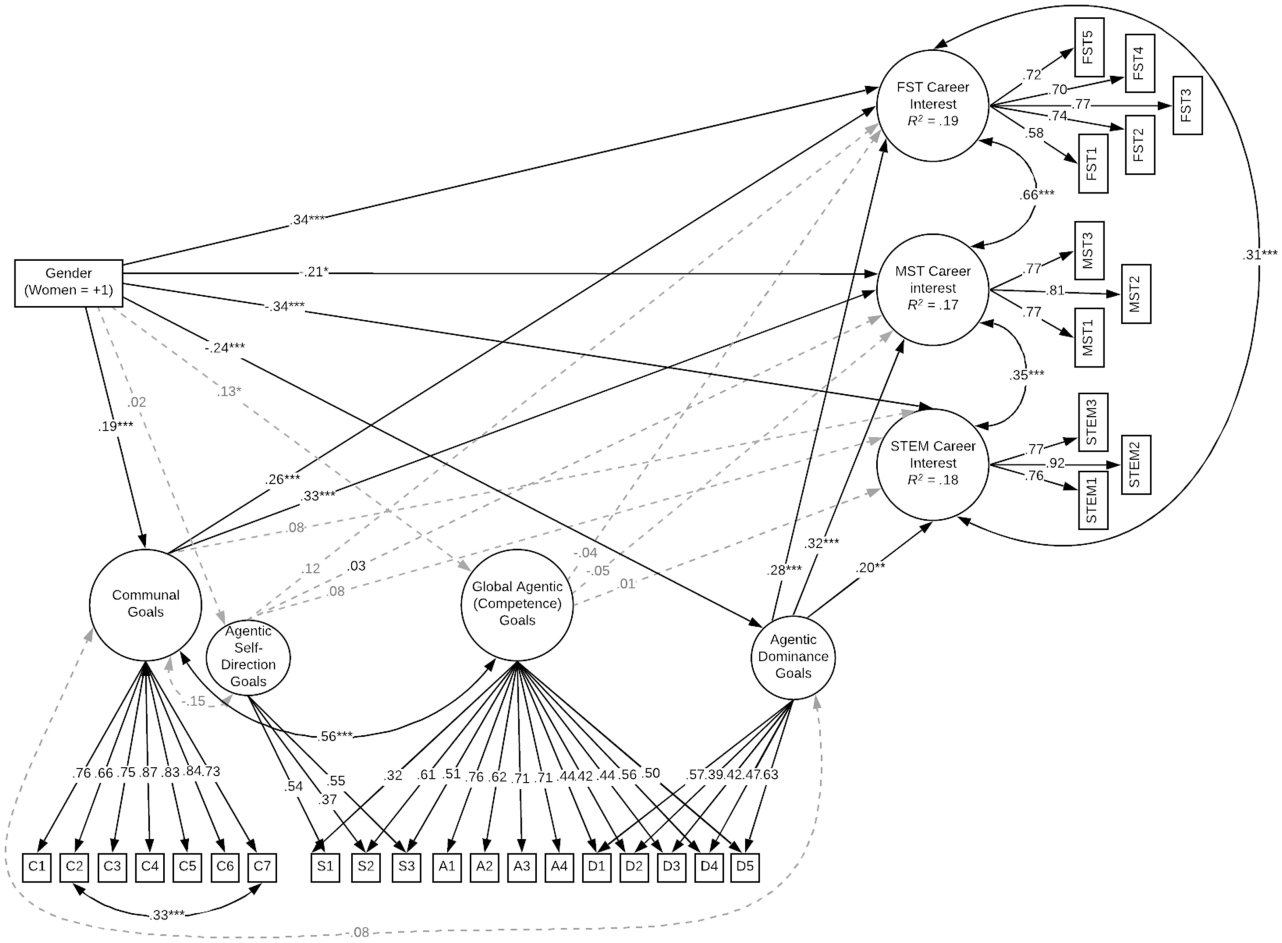
Structural bifactor model showing the relationships of gender to career interests *via* goal orientations. Direct effects of gender are denoted with Cohen’s *d*; all other estimates are standardized. MST, male stereotypic venture. FST, female stereotypic venture. Indirect effects indicate that dominance goal orientations explained men’s interest in MST, *β* = −0.08, *p* = 0.013, FST, *β* = −0.08, *p* = 0.006, and STEM ventures, *β* = −0.05, *p* = 0.04. In contrast, communal goal orientations explained women’s greater interest in FST ventures, *β* = 0.05, *p* = 0.032.

We estimated a structural model in which gender and goal orientations exhibited direct effects on venture interests, and gender exhibited indirect effects on venture interests *via* goal orientations (Figure 1), χ^2^(378) = 691.15, CFI = 0.926, TLI = 0.914, RMSEA = 0.046, 90%CI[0.040, 0.051], SRMR = 0.05. As expected, women had stronger communal goal orientations and weaker dominance goal orientations than did men. However, unlike Folberg et al. (2020), but consistent with Folberg et al. (2022) and Eagly et al. (2020), women (vs. men) had slightly stronger competence goal orientations. Gender differences also emerged in all three venture interests. Consistent with goal congruity theory (e.g., Diekman et al., 2010), women exhibited more interest in FST and less interest in MST and STEM ventures than did men. Thus, as expected, women and men preferred stereotype-consistent ventures.

Goal orientations were also directly related to venture interests although not always as expected. Communal goal orientations were associated with stronger FST venture interests, but also with stronger MST venture interests and not with STEM venture interests. Dominance goal orientations were associated with stronger interest in STEM and MST ventures, but also with stronger interest in FST ventures. The latter finding is inconsistent with goal congruity theory (but see Folberg et al., 2020). All three venture interest factors were highly correlated, perhaps reflecting societal assumptions that entrepreneurship requires dominance (e.g., Ahl, 2006). Individuals who have stronger dominance goal orientations may also be more career oriented. Consistent with Folberg et al. (2020), self-direction and global competence goal orientations were not associated with venture interests.

Finally, as expected, communal goal orientations partially explained women’s greater interest in FST venture interests, *β* = 0.05, *p* = 0.032, whereas dominance goal orientations partially explained men’s greater interest in MST, *β* = −0.08, *p* = 0.013, and STEM, *β* = −0.05, *p* = 0.046. Interestingly, dominance also explained men’s weaker FST venture interests, *β* = −0.06, *p* = 0.020. Consistent with our work examining general career interests (Folberg et al., 2020), self-direction and global competence goal orientations did not predict venture interests and, thus, could not explain gender differences in venture interests.

## 4. Discussion

Participants were more likely to ascribe agentic traits to successful entrepreneurs (e.g., Marlow, 2002; Ahl, 2006; Gupta and Fernandez, 2009), especially traits relating to dominance. We also replicated the bifactor model of goal orientations among aspiring entrepreneurs, which is consistent with other work showing the utility of agency and communion subdimensions (Hentschel et al., 2019; Folberg et al., 2020, 2022). As expected, women (vs. men) had stronger communal and weaker dominance goal orientations. Women were also more interested in starting FST ventures and less interested in starting MST and STEM ventures, consistent with work showing that women tend to exhibit stereotype-consistent venture interests (Wilhau and Karau, 2021). As expected, communal and dominance goal orientations partially accounted for differences in women’s and men’s venture interests. In contrast, global competence goals and self-direction goals did not.

### 4.1. The particular importance of dominance and communion

Women had somewhat stronger global competence goal orientations than did men, which is consistent with newer work (Hentschel et al., 2019; Eagly et al., 2020; Folberg et al., 2022). Neither global competence goals nor self-direction goals predicted venture interests, which may be surprising, as many individuals pursue entrepreneurial careers to have independence (Cromie, 1987; Wilhau and Karau, 2021). However, the U.S. is a highly individualistic culture that strongly values independence (Stephens et al., 2017), perhaps making self-direction a less useful predictor of venture interests in the U.S. than in other cultures (Folberg, 2020).

Dominance and communal goal orientations were, as expected (Folberg et al., 2020, 2022), the only dimensions that partially explained gender differences in venture interests. Thus, researchers who wish to measure or manipulate goal orientations—and specifically agentic goal orientations—should carefully consider how they operationalize them. Subdimensions of agency are not interchangeable, nor do global measures of agency accurately capture gender differences in subdimensions.

Dominance and communal goals also exhibited direct effects on venture interests in interesting and unexpected ways. Dominance goals were associated with stronger MST and STEM venture interests but also with greater FST interests, perhaps reflecting assumptions that even entrepreneurs starting stereotypically feminine ventures (e.g., salon/spa, childcare) need to exhibit dominance to compete for clients and resources. People who strongly endorse dominance goals may also be more generally career oriented (Folberg et al., 2020).

Communal goals were associated with interest in MST and FST careers, inconsistent with work suggesting that communal goals were negatively associated with MST careers (Diekman et al., 2010) and masculine environments (Folberg et al., 2020). Perhaps participants recognized that male-stereotypic ventures, such as auto-repair, are customer facing and require people to work with others (e.g., Abele et al., 2016). More generally, it suggests that although people may ascribe to entrepreneurs traits typically associated with men, they do not necessarily view entrepreneurship as incompatible with communal goals, posing interesting implications for entrepreneurial education and practice.

### 4.2. Implications for entrepreneurial education

Emphasizing that entrepreneurship is compatible with communal goals—even with respect to MST ventures—may increase women’s venture interests and encourage them to start a wider variety of ventures. Interventions designed to highlight the alignment between stereotypically masculine careers and communal goals increases STEM-specific (Boucher et al., 2017; Diekman et al., 2017) and general academic performance and persistence in among women and underrepresented college students (Boucher et al., 2017) and do not disadvantage men (Diekman et al., 2010).

These types of interventions may also be more successful than other practices, such as highlighting successful women who have succeeded in masculine entrepreneurial careers (Cochran, 2019), which tokenizes successful women in highly visible roles (Oakley, 2000). Further, highlighting women who succeed despite systemic discrimination does little to change the system and may result in initiatives that seek to “fix” women (e.g., to make them more confident or self-efficacious) rather than change systems (Diekman et al., 2017, 2020).

### 4.3. Practice implications

Explicitly highlighting the congruity between communal goals and entrepreneurship may change women’s perceptions of their ventures’ performance and investors’ choices of resource allocation. Investors and lenders might prioritize ventures that emphasize entrepreneurs’ communal qualities (e.g., serving community, helping others) to promote greater equity. Emphasizing communal qualities may lead to more “plodder” firms, which typically yield lower (financial) returns for investors than high-growth firms typically associated with men (Marlow, 2002). However, “plodder” firms may lead to greater community enrichment (Wilhau and Karau, 2021), which is also arguably a measure of success. Indeed, we question whether highly competitive, high-growth, high dominance firms are always desirable. STEM firms are replete with examples of high-valuation, high-growth firms, such as Uber (Isaac, 2017) and Theranos (Carreyrou, 2018), which prioritized profits over safety and health.

### 4.4. Limitations and future directions

The data were cross-sectional; thus, we cannot presume that goal orientations cause entrepreneurial interests. Venture interests were also not randomly selected from all possible MST, FST, and STEM careers. However, our model and findings are consistent with other work on goal congruity theory (Diekman et al., 2010, 2017; Folberg et al., 2020). Further, we did not assess the extent to which entrepreneurial careers are perceived as fulfilling agentic and communal goals. Future research might assess entrepreneurial goal affordances with respect to a wider variety of venture interests.

Our sample was also largely White, potentially limiting generalizability to other U.S. racial/ethnic groups. Both goal orientations and perceptions of STEM vary across cultures (Brown et al., 2018; Folberg, 2020; Folberg and Kaboli-Nejad, 2020). The effects of goal orientations among people of color, and whether a goal congruity framework might benefit marginalized groups underrepresented in entrepreneurship, remain important avenues for future research.

Finally, women encounter other types of systemic barriers, for example, disparate access to financial resources and fewer networking opportunities (Brush et al., 2019). Thus, changing perceptions of entrepreneurs must come hand in hand with other systemic changes.

## 5. Conclusion

Although individuals *perceive* entrepreneurship as requiring dominance, entrepreneurship is not inherently masculine and may satisfy communal goals, such as working with and caring for others. Further, women and men do not necessarily exhibit different levels of interest in entrepreneurship. Instead, they tend to prefer careers that align with socially prescribed gender roles, which are partially explained by dominance and communal goals. Thus, increasing women’s representation in entrepreneurship may require shifting perceptions of entrepreneurs and letting go of the myth that successful entrepreneurship requires dominance.

## Data availability statement

The datasets presented in this study can be found in online repositories. The names of the repository/repositories and accession number(s) can be found at: https://osf.io/dazy9/?view_only=229a10866c7b4afaace38dce69b3dfd8.

## Ethics statement

The studies involving human participants were reviewed and approved by the Institutional Review Board at the University of Nebraska Medical Center. Written informed consent for participation was not required for this study in accordance with the national legislation and the institutional requirements.

## Author contributions

AF drafted the manuscript and conducted the data analysis. TG assisted with the draft of the data analysis. XY provided feedback on drafts of the manuscript. CR provided feedback on drafts of the manuscript. LW collected the data. All authors contributed to the article and approved the submitted version.

## Conflict of interest

The authors declare that the research was conducted in the absence of any commercial or financial relationships that could be construed as a potential conflict of interest.

## Publisher’s note

All claims expressed in this article are solely those of the authors and do not necessarily represent those of their affiliated organizations, or those of the publisher, the editors and the reviewers. Any product that may be evaluated in this article, or claim that may be made by its manufacturer, is not guaranteed or endorsed by the publisher.
