# Supplementary material for: Viewing entrepreneurship through a goal congruity lens: The roles of dominance and communal goal orientations in women’s and men’s venture interests
Source: Front Psychol. 2023 Mar 22;14:1105550. doi: 10.3389/fpsyg.2023.1105550 (PMC10074595; doi:10.3389/fpsyg.2023.1105550)
Supplement: Supplementary file 1 [file Table_1.DOCX]

**Supplemental Materials**

To validate whether these careers were perceived as more commonly performed by women or men, a separate group of 50 participants (25 women, 25 men), recruited from Prolific Academic, were randomly assigned to estimate the percentage of women or the percentage of men in the 18 careers listed above on a 0 to 100% scale. Separate 2 (Target Gender) X 2 (Participant Gender) analyses of variance (ANOVAs) of FST, MST, and STEM careers revealed that participants perceived women as better represented in FST careers, *F*(1,46)=91.00, *p*<.001, η^2^=.67, and men as better represented in MST, *F*(1,46)=273.44, *p*<.001, η^2^=.86, and STEM careers, *F*(1,46)=132.98, *p*<.001, η^2^=.74. None of these effects depended on participant gender, nor were there any main effects of participant gender, *p*s>.089.

We also examined whether participants’ *perceptions* of each career aligned with the actual percentage of women and men in each career type. Data from the Bureau of Labor Statistics (2019) confirmed that construction (3.5% women), auto repair (1.0% women), lawncare/landscaping (6.5% women), and financial advising (34.0% women) were more commonly occupied by men than women, as were computer/cell phone repair (1.5% women), IT consulting (26.4% women), and software development (19.4% women), which we categorized as STEM careers. In contrast, florist (81.5% women), childcare (94.8% women), salon/spa (90.8% women), cleaning services (88.3% women), interior design (84.0% women) and event planning (78.7% women) were more commonly performed by women than men. Further, participants’ percentage estimates of women and men in each career were nearly perfectly correlated with the actual numbers, *r*s(16)=.96, *p*s<.001, for women and men, respectively. Thus, our work aligns with past studies, suggesting that individuals’ stereotypes are often accurate at the group level (Froelich et al., 2020; Author, 2002).

**Table 1**

*Entrepreneurial Aspirations/Career Areas Listed by Gender*

| Career | men | women | Career | men | women | Career | men | women |
| --- | --- | --- | --- | --- | --- | --- | --- | --- |
| Crafts store | 4 | 21 | App development | 0 | 3 | Travel | 1 | 0 |
| Consulting business | 12 | 4 | Construction | 1 | 2 | Tutoring | 0 | 1 |
| Restaurant | 9 | 7 | Content creation | 1 | 2 | Wildlife sanctuary | 1 | 0 |
| Web-based industry | 9 | 7 | Engineering | 3 | 0 | Winery | 1 | 0 |
| Fashion | 1 | 13 | Film | 2 | 1 | Yoga | 0 | 1 |
| Coffee shop | 3 | 10 | Research | 2 | 1 | UPS franchisee | 0 | 1 |
| Counseling services | 3 | 7 | Bar | 2 | 0 | Waste disposal | 0 | 1 |
| Bakery | 3 | 6 | Bookkeeping | 0 | 2 |  |  |  |
| Finance | 9 | 0 | Event-planning | 0 | 2 |  |  |  |
| Graphic design | 3 | 6 | Grocery | 1 | 1 |  |  |  |
| Retail | 3 | 6 | Journalism | 0 | 2 |  |  |  |
| Technology start-up | 6 | 3 | Pharmaceuticals | 2 | 0 |  |  |  |
| Art | 3 | 5 | Sex work | 1 | 1 |  |  |  |
| Healthcare | 1 | 7 | Tourism | 1 | 1 |  |  |  |
| Pets services | 2 | 6 | Youth programs | 2 | 0 |  |  |  |
| Real estate | 7 | 0 | Veterinary practice | 1 | 1 |  |  |  |
| Software | 6 | 1 | Catering | 0 | 1 |  |  |  |
| Car/auto repair | 5 | 1 | Childcare | 0 | 1 |  |  |  |
| Fitness/sports | 6 | 0 | Cybersecurity | 1 | 0 |  |  |  |
| Cannabis dispensary | 2 | 3 | Data science/GIS business | 1 | 0 |  |  |  |
| Computer repair | 5 | 0 | Deli | 1 | 0 |  |  |  |
| Toy store | 4 | 1 | Drop shipping | 1 | 0 |  |  |  |
| Writer | 3 | 2 | Education | 1 | 0 |  |  |  |
| Beauty | 1 | 3 | Entertainment | 1 | 0 |  |  |  |
| Book store | 3 | 1 | Farm | 1 | 0 |  |  |  |
| Brewery | 3 | 1 | Franchise-owner | 1 | 0 |  |  |  |
| Cleaning | 1 | 3 | Funeral home | 0 | 0 |  |  |  |
| Food truck | 4 | 0 | Furniture store | 0 | 1 |  |  |  |
| Game development | 4 | 0 | Landscaping | 1 | 0 |  |  |  |
| IT consulting | 3 | 1 | Lobbying | 0 | 0 |  |  |  |
| Jewelry making | 0 | 4 | Manufacturing | 1 | 1 |  |  |  |
| Legal services | 1 | 3 | Outdoor recreation | 0 | 0 |  |  |  |
| Marketing | 2 | 2 | Services for Special Needs Adults | 1 | 0 |  |  |  |
| Music | 3 | 1 | Salon | 0 | 1 |  |  |  |
| Accounting firm | 2 | 1 | Solar energy systems | 1 | 0 |  |  |  |

*Note.* Fifteen participants did not indicate their entrepreneurial interests in response to the open-ended question.

**Table 2**

*Item-Level Descriptive Statistics*

| Item | *M* | *SD* | Skewness | Kurtosis |
| --- | --- | --- | --- | --- |
| C1. Serving humanity | 5.31 | 1.44 | -1.03 | 1.06 |
| C2. Working with people | 4.95 | 1.62 | -0.66 | -0.10 |
| C3. Attending to others | 4.95 | 1.52 | -0.73 | 0.25 |
| C4. Helping others | 5.61 | 1.37 | -1.30 | 1.88 |
| C5. Serving community | 5.31 | 1.48 | -1.07 | 1.05 |
| C6. Caring for others | 5.34 | 1.45 | -0.90 | 0.72 |
| C7. Connection with others | 5.31 | 1.48 | -0.93 | 0.72 |
| A1. Achievement | 5.50 | 1.40 | -1.27 | 1.83 |
| A2. Success | 5.73 | 1.22 | -1.30 | 2.41 |
| A3. Demonstrating skill/competence | 5.71 | 1.37 | -1.42 | 2.27 |
| A4. Mastery | 5.46 | 1.37 | -0.91 | 0.85 |
| D1. Power | 3.62 | 1.81 | 0.06 | -1.06 |
| D2. Self-promotion | 4.38 | 1.63 | -0.23 | -0.49 |
| D3. Competition | 4.23 | 1.62 | -0.29 | -0.53 |
| D4. Recognition | 4.68 | 1.60 | -0.52 | -0.24 |
| D5. Status | 4.19 | 1.73 | -0.14 | -0.86 |
| D6. Financial rewards | 5.63 | 1.32 | -1.24 | 1.91 |
| S1. Individualism | 5.29 | 1.48 | -0.90 | 0.66 |
| S2. Self-direction | 5.55 | 1.32 | -1.04 | 1.36 |
| S3. Independence | 5.86 | 1.22 | -1.35 | 2.32 |
| S4. Focus on the self | 5.12 | 1.44 | -0.66 | 0.23 |
| STEM1. Computer/cell phone repair | 2.50 | 1.89 | 1.01 | -0.25 |
| STEM2. IT Consulting | 2.78 | 2.07 | 0.73 | -0.94 |
| STEM3. Software development | 2.88 | 2.07 | 0.62 | -1.04 |
| MST1. Construction | 1.89 | 1.52 | 1.72 | 2.08 |
| MST2. Auto repair | 1.68 | 1.26 | 2.02 | 3.32 |
| MST3. Lawn care/landscaping | 1.97 | 1.48 | 1.44 | 1.01 |
| MST4. Financial advising | 2.60 | 1.81 | 0.78 | -0.67 |
| FST1. Child care | 2.17 | 1.63 | 1.29 | 0.57 |
| FST2. Salon/spa | 2.03 | 1.63 | 1.51 | 1.20 |
| FST3. Interior design | 2.56 | 1.81 | 0.83 | -0.65 |
| FST4. Event planning | 2.74 | 1.92 | 0.66 | -0.97 |
| FST5. Florist | 2.19 | 1.63 | 1.24 | 0.50 |
| FST6. Cleaning services | 2.23 | 1.71 | 1.24 | 0.35 |

*Note. N* = 349-351.

**Planned Analyses**

We developed our measurement model by first estimating separate models for goal orientations and venture interests. As we developed venture interest items for this study, we estimated exploratory structural equation models (ESEMs) to test the hypothesized three-factor structure of venture interests and assessed the relative fit of alternative one-, two-, and three-factor solutions. Confirmatory factor analyses (CFAs) were used to confirm the first-order factor structure of goal orientations suggested by Authors et al. (2020) and to assess the fit of a model including the first-order goal orientations and venture interest latent factors.

Tests of nested models (e.g., alternative factor structures) were assessed using recommendations from Chen (2007). Decrements in the CFI of greater than .01 and increases in the RMSEA of greater than .015 indicated significant degradations of model fit. Our a priori criteria for adequate factor loadings considered primary loadings that were less than .40 and secondary loadings that exceeded .30 to be potentially problematic. Finally, we used McDonald’s (1999) omega to evaluate the reliability of each latent factor.

We then estimated a bifactor CFA of goal orientations following the bifactor model specified by Authors et al. (2020). We allowed all items assessing communal goal orientations s to load onto a latent factor assessing communal goal orientations. All agentic goal orientation items were allowed to load onto a global latent factor assessing global agentic goal orientations, as well as their specific subdimension. For bifactor analyses, all items were allowed to freely load onto their respective latent factors and factor variances were fixed to 1 for purposes of model identification. Communal goals were allowed to covary with global agentic goal orientations and each agentic goal orientation subdimension. However, covariances between the global agentic goal orientation factor and each subdimension as well as covariances among the specific agentic goal orientations subdimensions were fixed to zero.

**Measurement Model of Venture Interests**. We conducted ESEM analyses of venture interests, estimating one through three factor structures of the 13 items assessing venture interests. The one-factor, χ^2^(65) = 672.53, CFI = .557, TLI = .469, RMSEA = .164, 90%CI [.15, .18], SRMR = .13, and two-factor, χ^2^(53) = 244.51, CFI = .86, TLI = .795, RMSEA = .102, 90%CI [.09, .12], SRMR = .058, solutions exhibited poor fit. However, the hypothesized three-factor model of MST, FST, and STEM venture interests exhibited excellent global fit, χ^2^(42) = 87.86, CFI =.967, TLI = .938, RMSEA = .056, 90%CI [.04, .07], SRMR = .03. One residual correlation exceeded .14; all other residual correlations were below .09, suggesting generally good local fit. However, Financial advisor (MST4) exhibited an inadequate primary loading on MST ventures (λ = .19) and Cleaning services exhibited a problematic cross-loading on MST ventures (λ = .32). In retrospect, these items might be influenced by both the gender and socioeconomic status of individuals who are perceived as likely to pursue these jobs. We subsequently dropped the two items from the model.

The resulting three-factor model exhibited excellent global fit, χ^2^(25) = 42.90, CFI =.984, TLI = .965, RMSEA = .045, 90%CI [.02, .07], SRMR = .02. Residual correlations did not exceed .10, indicating excellent local fit. All items loaded onto their primary factors above .555. Secondary loadings did not exceed .172. Correlations among factors ranged from .211 to .528.

**First-Order CFA of Goal Orientations.** We estimated a four-factor model of the 21 goal orientation items reflecting communal goals and agentic dominance, competence, and self-direction goals following the factors identified by Authors et al. (2020). This four-factor model exhibited poor global fit, c2(183) = 422.26, CFI =.892, TLI = .876, RMSEA = .061, 90%CI [.054, .069], SRMR = .066, although all items exhibited adequate loadings λ > .569. Examinations of residual correlations and modification indices suggested allowing Financial rewards (D6) to load onto both agentic dominance and competence goals and allowing Focus on the self (S4) to load onto both dominance and competence goals. Notably, Authors et al. (2020) found that these two items loaded unreliably on their primary factors across two studies, suggesting that they may not be reliable indicators of agentic goals. As such, we dropped them from the measurement model. This trimmed model exhibited adequate fit, χ^2^(146) 305.226, CFI = .917, TLI = .903, RMSEA = .056, 90%CI [.047, .065], SRMR = .06. However, eight residual correlations exceeded .10, and one exceeded .20, suggestion potential local mis-fit. At the suggestion of modification indices, we, therefore allowed the errors between Helping others (C4) and Caring for others (C6) to covary.

The resulting final measurement model exhibited adequate global fit, χ^2^ (145) = 282.44 CFI =.928, TLI = .916, RMSEA = .052, 90%CI [.043, .061], SRMR = .061. All primary loadings were strong and significant for items assessing communal goals, (C1-C7; λ = .653-.876, *M* = .778), agentic dominance goals (D1-D5; λ = .578-.771, *M* = .674), agentic competence goals (A1-A4; λ = .621-.755, M = .693), and agentic self-direction goals (S1-S3; λ = .536-.758, M = .670). Residual correlations did not exceed an absolute value of .216. Further 98.8% of residual correlations were below an absolute value of .20, and 87.7%% of residual correlations were below the threshold of .10, indicating generally adequate local fit.

**Bifactor CFA of Goal Orientations.** We then estimated bifactor CFA model of goal orientations, in which all communal goals loaded onto their primary factor and all agentic goal orientation items were allowed to load onto their respective dominance, competence, and self-direction subdimensions, as well as a global agentic goal orientation factor. This initial bifactor model failed to converge because of a negative residual variance for the item success (AX), which also occurred in Folberg et al. (2020, Study 1). We fixed the residual variance to equal zero and estimated the model, which exhibited adequate global fit, χ^2^(136) = 246.64, CFI = .942, TLI = .928, RMSEA = .048, 90%CI [.039, .058], SRMR = .054. However, two of the four agentic competence items failed to load on their primary factors, *p*s > .148, and of the two significant loadings, one was positive and one was negative. Following Authors et al.. (2020), we, therefore, removed the agentic competence goal subdimension, and allowed all agentic competence goal items to load only onto the global agentic goals factor.

Following Authors et al. (2020), we dropped the specific competence goal orientation factor, as agentic competence goal orientations were indistinguishable from the global agentic goal orientations. Thus, we refer to the global factor as global competence goal orientations. This model exhibited adequate global fit, χ^2^(140) = 267.97, CFI =.933, TLI = .919, RMSEA = .051, 90%CI [.042, .061], SRMR = .057. Residual correlations did not exceed .211; 99.4% of residual correlations were below and absolute value of .20, and 87.7 % of residual correlations were below an absolute value of .10, suggesting evidence of generally good local fit. We also examined ωH for the global competence goal orientations factor and ωHS for the agentic dominance and competence goal orientation domain-specific factors to determine the appropriateness of the bifactor model. ωH = .75, which is below the cut-off for a unidimensional model (Rodriguez et al., 2016). In addition, ωHS = .43 and .37 for agentic dominance and self-direction goal orientations, respectively, suggesting that they accounted for a non-negligible amount of variance in their respective items. In sum, Hypothesis 2 was supported; agentic and communal goal orientations comprised a unidimensional communal goal orientation measure, a global measure of competence goal orientations and specific subdimensions of agentic dominance and self-direction goal orientations.

Communal goal orientation items again loaded strongly and significantly on their primary factor (λ = .653-.877, *M* = .778). Items assessing agentic dominance (λ = .396-.646, *M* = .494) and agentic self-direction goal orientations (λ = .369-.549, *M* = .486) loaded significantly onto their respective subdimensions. Further, all agentic goal orientation items loaded strongly and significantly onto the global competence goal orientation factor (λ = .317-.706, *M* = .544).

Recall that to estimate the bifactor model all correlations among the global agentic goal orientation factor and its subdimensions were fixed to zero. Consistent with Authors et al. (2020) communal goal orientations were strongly and positively associated with global agentic competence goal orientations, *r* = .565, *p* < .001, weakly associated with lower agentic dominance goal orientations, *r* = -.17, *p* = .019, and unassociated with agentic self-direction goal orientations, *p* = .167.

**Full Measurement Model.** We then added the 11 items assessing career interests and allowed them to load onto their respective MST, FST, and STEM factors. (See Table 3 in the main text of the paper for factor loadings, correlations among factors, and estimates of McDonald’s [1999] omega.) This model exhibited adequate global fit, χ^2^(378) = 633.584, CFI =.931, TLI = .921, RMSEA = .044, 90%CI [.038, .050], SRMR = .053. Residual correlations did not exceed an absolute value of .211. Approximately 99.8% of residual correlations were below an absolute value of .20, and 93.3% of correlations were below and absolute value of .10, suggesting good evidence of local fit.

**Table 3**

*Means by Gender and Correlations Among Composite Measures*

| Measure | Women  (*n* = 175) | Men  (*n* = 176) | 1 | 2 | 3 | 4 | 5 | 6 | 7 |
| --- | --- | --- | --- | --- | --- | --- | --- | --- | --- |
| Goal Orientations |  |  |  |  |  |  |  |  |  |
| 1. Communal | 5.49  (1.19) | 5.03  (1.18) | *.91* | .42* | .29*** | .27*** | .06 | .23*** | -.02 |
| 2. Global Competence | 5.03  (0.95) | 5.00  (0.94) |  | *.86* | .87*** | .68*** | .12* | .19*** | .10 |
| 3. Agentic Dominance | 4.12  (1.23) | 4.33  (1.30) |  |  | *.81* | .33*** | .20*** | .20*** | .17*** |
| 4. Agentic Self-direction | 5.65  (1.07) | 5.48  (1.09) |  |  |  | *.73* | .00 | .11* | .02 |
| Venture Interests |  |  |  |  |  |  |  |  |  |
| 5. MST | 1.55  (1.07) | 2.14  (1.29) |  |  |  |  | *.82* | .46*** | .39*** |
| 6. FST | 2.70  (1.45) | 1.98  (1.09) |  |  |  |  |  | *.83* | .20*** |
| 7. STEM | 2.10  (1.55) | 3.34  (1.77) |  |  |  |  |  |  | *.85* |

*Note. N =* 351. Standard deviations are in parentheses. Cronbach’s alphas are on the diagonal. MST = male-stereotypic. FST = female-stereotypic. STEM = science, technology, engineering, and mathematics.

**p* < .05. ***p* < .01. ****p* < .001.

**References**

Author (2002). *Manuscript blinded for review*

Chen, F. F. (2007). Sensitivity of goodness of fit indexes to lack of measurement invariance. *Structural Equation Modeling, 14*(3), 464–504. <https://doi.org/10.1080/10705510701301834>

Froehlich, L., Olsson, M. I., Dorrough, A. R., & Martiny, S. E. (2020). Gender at work across nations: Men and women working in male‐dominated and female‐dominated occupations are differentially associated with agency and communion. *Journal of Social Issues*, *76*(3), 484-511. https://doi.org/10.1111/josi.12390
